# Supplementary material for: Ultrastable microwave and soliton-pulse generation from fibre-photonic-stabilized microcombs
Source: Nat Commun. 2022 Jan 19;13:381. doi: 10.1038/s41467-022-27992-8 (PMC8770478; doi:10.1038/s41467-022-27992-8)
Supplement: Supplementary file 1 — Supplementary Information [file 41467_2022_27992_MOESM1_ESM.pdf]

Supplementary Information for
“Ultrastable microwave and soliton-pulse generation from fibre-photonic-stabilized microcombs”

Dohyeon Kwon[1,#], Dongin Jeong[2,#], Igju Jeon[1], Hansuek Lee[2,3,*], and Jungwon Kim[1,*]

[1]*School of Mechanical and Aerospace Engineering, Korea Advanced Institute of Science and Technology (KAIST), Daejeon 34141, Korea*
[2]*Graduate School of Nanoscience and Technology, Korea Advanced Institute of Science and Technology (KAIST), Daejeon 34141, Korea*
[3]*Department of Physics, Korea Advanced Institute of Science and Technology (KAIST), Daejeon 34141, Korea*

[#]*These authors contributed equally to this work.*
* *e-mails: hansuek@kaist.ac.kr; jungwon.kim@kaist.ac.kr*

Supplementary Note 1: Micro-comb generation

Pump-cavity detuning lock. The comb power detected by PD in Supplementary Fig. 1 changes when the laser frequency is swept over time. We set the locking point by the input offset voltage of the loop filter. The error signal in the loop filter corresponds to the difference between the set voltage (V_{ref}) and the comb power (V_{in}), that is, $V_{error} = V_{in} - V_{ref}$. If the comb power fluctuates, the laser frequency is modulated. The feedback is conducted to maintain V_{error} to zero. We summarized this process in Supplementary Fig. 2.

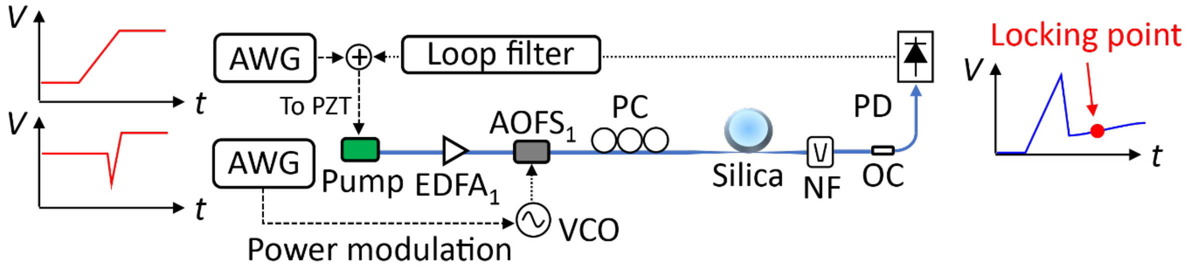

Supplementary Figure 1 | Microcomb generation. Schematic of microcomb generation. To maintain the soliton mode from thermal destabilization, the pump-cavity detuning is locked via feedback control applied to the laser PZT. AWG, arbitrary waveform generator; AOFS$_1$, acousto-optic frequency shifter; VCO, voltage-controlled oscillator; PC, polarization controller; NF, notch filter; OC, optical coupler; PD, photodetector.

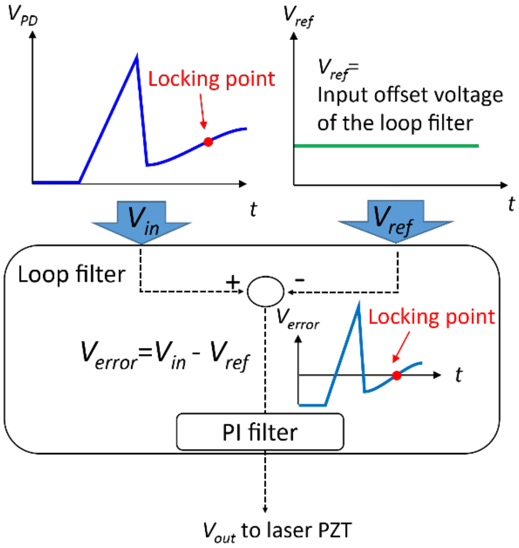

Supplementary Figure 2 | Pump-cavity detuning lock. Schematic of the pump-cavity detuning lock. V_{in}, photodetected comb power; V_{ref}, set voltage for the pump-cavity detuning lock; V_{error}, resulting error voltage for the pump-cavity detuning lock.

Finding the quiet point. The detuning between the pump and cavity is highly critical to ensure their noise performance. The repetition rate is determined not only by the free spectral range of the resonator but also by the Raman-self frequency shift[1] and dispersive recoil[2] as a function of the pump-cavity detuning ($\delta\omega$)[3]. Therefore, the repetition rate can be rewritten as $f_{\mathrm{rep}}=D_1/2\pi+(\Omega_{\mathrm{Raman}}(\delta\omega)+\Omega_{\mathrm{Recoil}}(\delta\omega))\times D_2/2\pi D_1$, where $D_1/2\pi$ is the free-spectral range of the resonator and $D_2/2\pi$ is the second-order dispersion. By carefully changing the pump-cavity detuning, the contributions of the Raman self-frequency shift and dispersive recoil to the phase noise can be minimized. In our experimental setup (Supplementary Fig. 3**a**), the pump-cavity detuning is adjusted by changing the input reference level of the servo loop filter (see Supplementary Fig. 2), and the phase noise is monitored by the phase noise analyser.

The microcomb shows the lowest timing jitter when the pump-cavity detuning is 16 MHz. The microwave phase noise PSDs near the quiet point are plotted (Supplementary Fig. 3**b**). The phase noise below 100-kHz Fourier frequency changes when the pump-cavity detuning

changes because the transfer function of the phase noise from the pump-cavity detuning works as a first-order lowpass filter whose cut-off frequency is the cavity decay rate[3]. In other words, since the phase noise at high Fourier frequencies is determined by the Q-factor of the resonator, it is highly desirable to use an ultrahigh Q-factor resonator such as a silica resonator to minimize the phase noise at high Fourier frequencies.

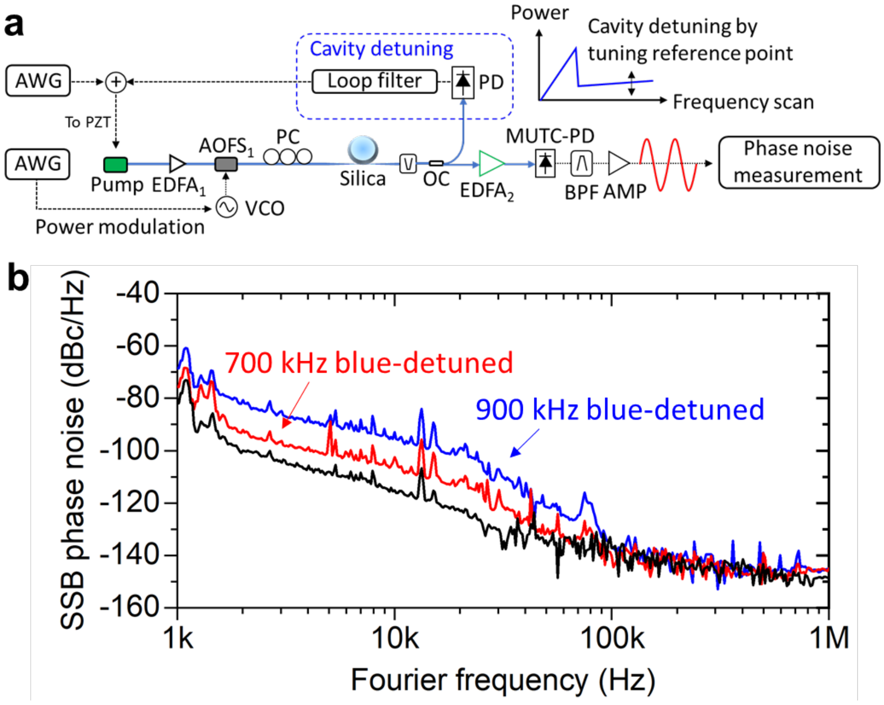

Supplementary Figure 3 l Noise performance near the quiet point. Experimental setup and noise of microcombs. **a,** Experimental setup for finding and maintaining the quiet point. AWG, arbitrary waveform generator; PC, polarization controller; EDFA, Er-doped fibre amplifier; MUTC-PD, modified uni-travelling carrier photodiode; BPF, RF bandpass filter; AMP, RF amplifier. **b,** Phase noise of the free-running microcomb near the quiet point. Red and blue curves are 700 kHz and 900 kHz blue-detuned from the black curve, respectively.

Supplementary Note 2: Fibre delayline-based repetition-rate stabilization

Bandpass filtering conditions. The frequency spacing and filtering bandwidth of bandpass filters are carefully selected. In our scheme, we need at least a few mW for effective self-heterodyning with sufficient SNR, therefore, EDFA is required after the bandpass filters. If we

filter a single comb-line, the signal power is too low (a few μW) to achieve this level with sufficient SNR due to the amplified spontaneous emission (ASE) noise of the EDFA. Therefore, we need broader bandwidth filters for higher input power to the EDFA. In the experiment, we used 2-nm bandwidth bandpass filters.

As shown in the error signal formula, $\delta[\tau\times(m\text{-}n)f_{\text{rep}}]$, the phase detection sensitivity scales with both the delay time τ and the frequency separation $(m\text{-}n)f_{\text{rep}} = \nu_m - \nu_n$. While it is clear that larger (m-n) will enable higher detection sensitivity, if the frequency separation is too large beyond the optical bandwidth of soliton spectrum, the filtered power as well as SNR will decrease. As shown in Supplementary Fig. 4, the combination of 1540 nm and 1560 nm provides a reasonable performance with ~11 dB attenuation from the peak. When we used larger separation (e.g., 1530 nm and 1570 nm), the usable power was too low and lowered the SNR of the beating signal.

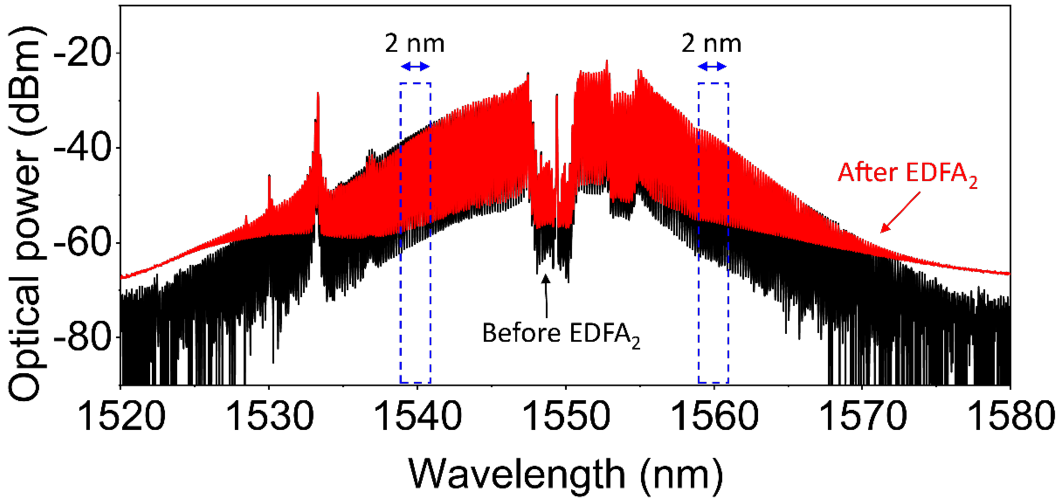

Supplementary Figure 4 I Optical spectrum before and after the C-band EDFA. Optical spectrum of the soliton is displayed. Blue dashed boxes denote the filtered spectrum for the repetition-rate stabilization.

High-speed repetition-rate tuning mechanism. Supplementary Figure 5 shows the schematic

for the high-speed repetition-rate tuning and broadband repetition-rate stabilization based on the customized voltage-controlled oscillator (VCO).

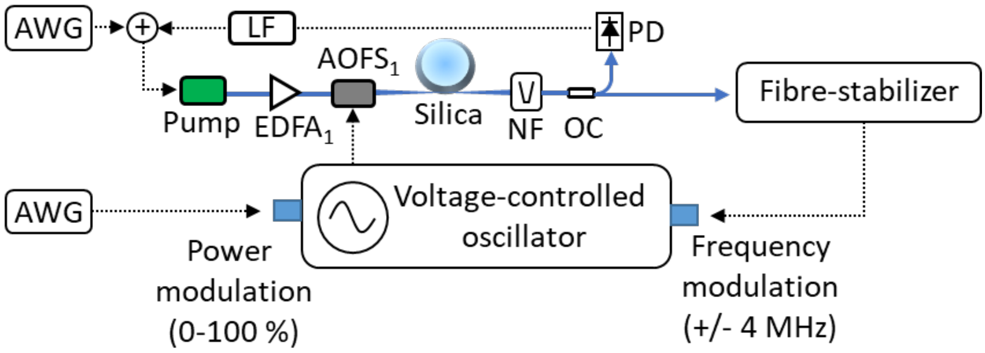

Supplementary Figure 5 | Schematic for the repetition-rate stabilization. Repetition rate is stabilized by the external frequency modulation via voltage-controlled oscillator (VCO) applied to AOFS$_1$.

Crosstalk between soliton mode-locking and repetition-rate stabilization. In our experiment, two feedback loops (pump-cavity detuning lock and repetition-rate lock) are applied simultaneously. The pump-cavity detuning is locked via a piezoelectric transducer (PZT) of the pump laser to maintain the soliton power since the soliton power is the function of the pump-cavity detuning[4] (see Supplementary Fig. 1). At the same time, the repetition-rate noise discriminated by a fibre delayline is suppressed by the external frequency modulation via a VCO applied to the acousto-optic frequency shifter (AOFS$_1$ in Supplementary Fig. 5). The effect of the repetition-rate stabilization on soliton-mode stabilization is quantitatively assessed as described below.

In order to quantify the conversion coefficient of pump-frequency to pump-intensity, we modulated the pump frequency by applying a 2-kHz sinewave to the VCO as shown in Supplementary Fig. 6. Then, we measured the pump frequency noise PSD[5] and the relative intensity noise (RIN) PSD of the comb output simultaneously. By comparing the 2-kHz modulation peaks of the pump frequency noise PSD and the comb RIN PSD, the pump

frequency noise-to-comb RIN conversion coefficient (α) [in (1/Hz)/(Hz^2/Hz)=1/Hz^2 unit] is obtained as 10^{-20} [1/Hz^2].

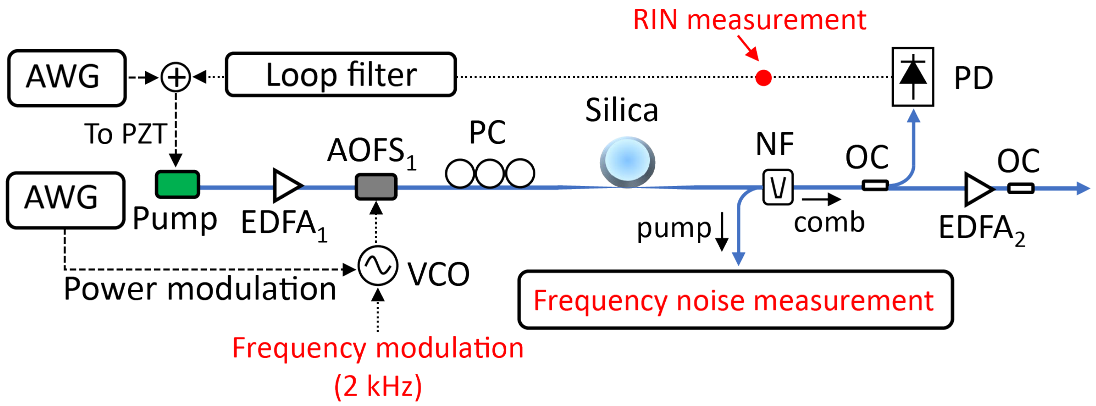

Supplementary Figure 6 I Experimental setup for obtaining the coefficient of the external pump frequency modulation and relative intensity noise. Pump frequency is modulated with 2 kHz via a voltage-controlled oscillator (VCO) applied to an acousto-optic frequency shifter ($AOFS_1$) in order to calculate the coefficient (α) between the pump frequency noise and the relative intensity noise of the microcomb (comb RIN).

Then, we measured the voltage noise PSD of the input signal to the VCO when the repetition-rate control loop is closed (Supplementary Fig. 7**a**). By dividing this measured PSD by the sensitivity of the used VCO (0.8 MHz/V), we can convert the measured PSD to the pump frequency noise PSD of the stabilized repetition-rate control loop (Supplementary Fig. 7**b**). Finally, by using the pump frequency noise-to-comb RIN conversion coefficient ($\alpha=10^{-20}$ [1/Hz^2]), we can convert the pump frequency noise PSD into the equivalent comb-RIN PSD. As shown in Supplementary Fig. 7**c**, the equivalent RIN induced by the repetition-rate control is >20 dB lower than the measured comb-RIN, which shows that the repetition-rate stabilization does not hamper the soliton-mode locking, and both control loops can operate simultaneously.

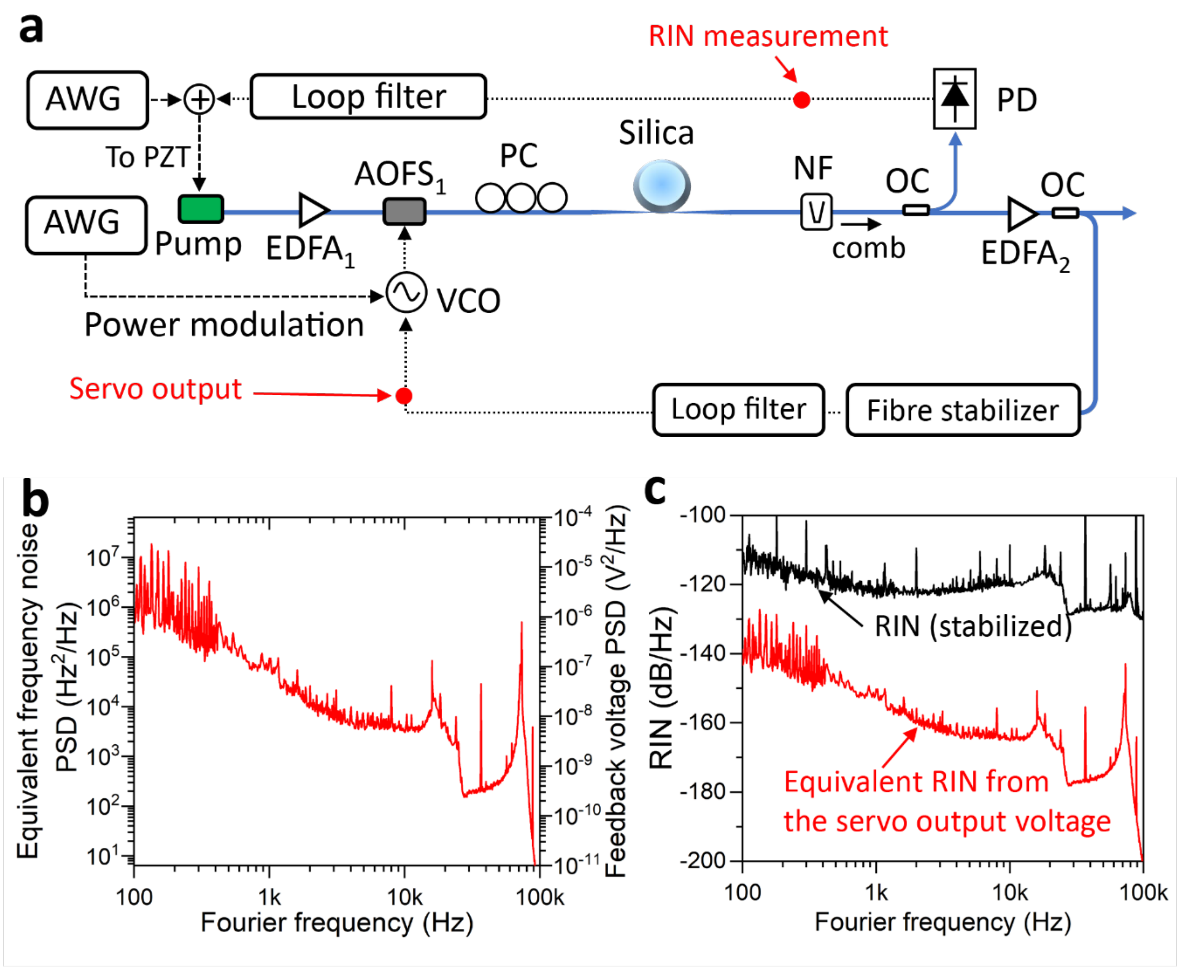

Supplementary Figure 7 | Experimental setup and results of the equivalent RIN contribution from the servo output voltage. a, Experimental setup. Servo output voltage PSD and comb RIN are measured when repetition-rate is stabilized by a fibre stabilizer. **b,** Equivalent pump frequency noise PSD and measured servo output voltage PSD (i.e., feedback voltage PSD) when the repetition rate is stabilized. **c,** Equivalent RIN from the servo output voltage PSD and the measured comb RIN when the repetition rate is stabilized.

Supplementary Note 3: Noise analysis

Phase noise analysis of the free-running and stabilized microcombs. Timing jitter of free-running microcombs is fundamentally limited by the quantum noise[6,7] and the intermode thermal noise[8,9] (i.e., thermorefractive noise). By using the comb parameters, we plotted the projected quantum noise (curve (vi)) and thermorefractive noise (curve (vii)) along with the

measured free-running (curve (i)) and stabilized (curves (ii)-(iv)) comb phase noise in Supplementary Fig. 8.

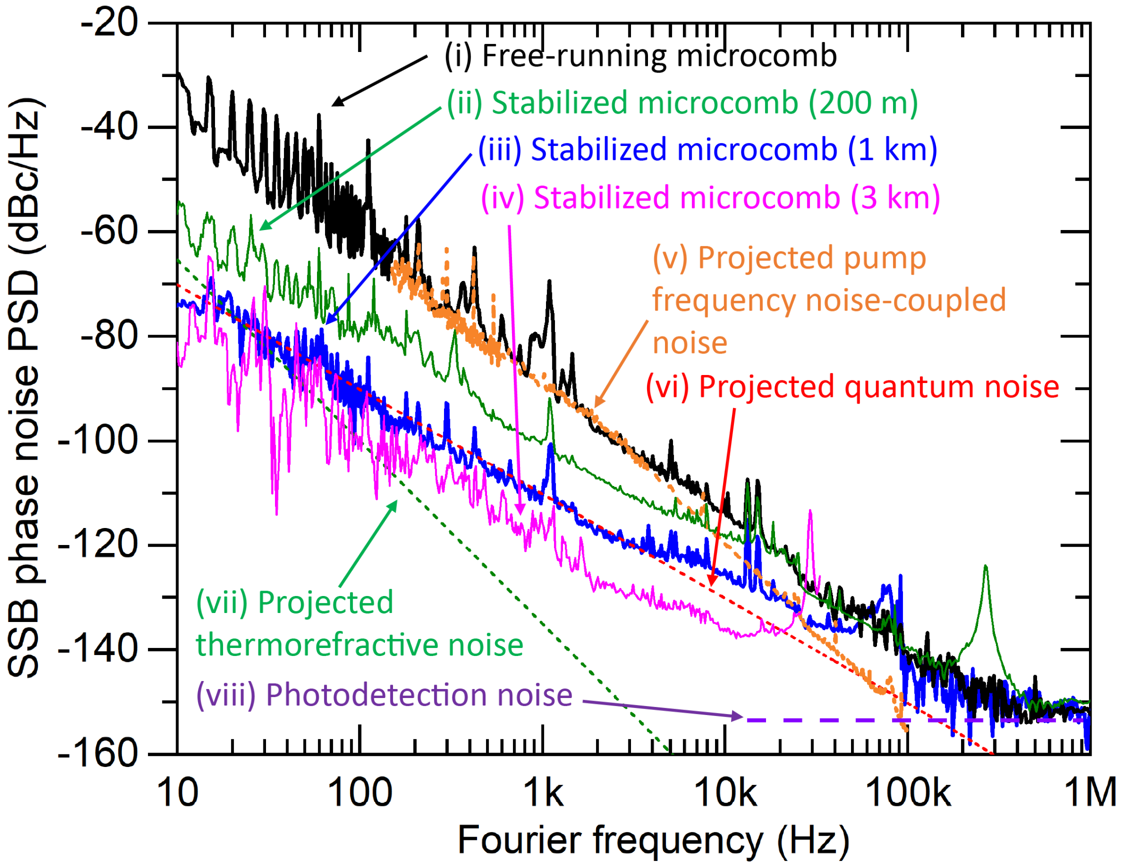

Supplementary Figure 8 l Noise limiting factors for the microcomb. (i) Measured phase noise of the free-running microcomb. (ii) Measured phase noise of the stabilized microcomb (using 200-m fibre link). (iii) Measured phase noise of the stabilized microcomb (using 1-km fibre link). (iv) Measured phase noise of the stabilized microcomb (using 3-km fibre link). (v) Projected pump frequency noise-coupled phase noise with free-parameters. (vi) Projected quantum noise[6,7]. (vii) Projected thermorefractive noise[8,9]. (viii) Photodetection white noise floor in our system.

Regarding free-running jitter, the high-frequency phase noise closely approaches the quantum noise within 10 dB from ~30 kHz offset frequency and is limited by photodetection white noise floor (curve (viii)) from 300 kHz offset frequency. In the lower offset frequency (<30 kHz), the phase noise is limited by more technical noise. Although it is still a preliminary

stage and beyond the scope of this paper, we believe that the pump frequency noise-originated jitter, which is coupled by the microresonator dispersion, is the dominant noise source (projected fitting curve (v) using free parameters).

Regarding the stabilized jitter, the free-running jitter (which is limited by the quantum noise) is the limiting factor for high frequency beyond the stabilization bandwidth (e.g. ~100 kHz for 1-km fibre case). Inside the stabilization bandwidth, it is mostly limited by the fibre delay itself (i.e., -20 dB/dec phase noise slope down to 10 Hz), not limited by the fundamental noise (such as quantum noise or thermorefractive noise) of the free-running combs. The main reason for the residual white noise floor (in frequency noise PSD) is the Rayleigh scattering RIN. While the Rayleigh scattering PSD scales with the delay length[10], the enhanced detection sensitivity (which scales to the delay length) improves the phase noise PSD by the square of the delay length. As a result, when the Rayleigh scattering RIN scaling and timing detection sensitivity scaling are combined, the resulting frequency noise PSD floor inversely scales with the fibre delay length. As shown by curves (ii) to (iv), where the delay fibre length is increased from 200 m to 3 km, locking to a longer fibre delay indeed enables lower phase noise inside the locking bandwidth.

We also assessed the impact of RIN-converted phase noise in the photodetection. We measured the amplitude-to-phase conversion coefficient[11] (APC) of the used photodiode by modulating the comb intensity and measuring the 22-GHz phase noise after photodetection. At 10, 100 and 200 kHz Fourier frequency, the APC was measured to be 8 rad/%. From the measured APC, the projected phase noise due to the comb RIN is plotted in Supplementary Fig. 9. As shown here, the RIN-converted photodetection noise is below the stabilized performance and does not limit the measurement.

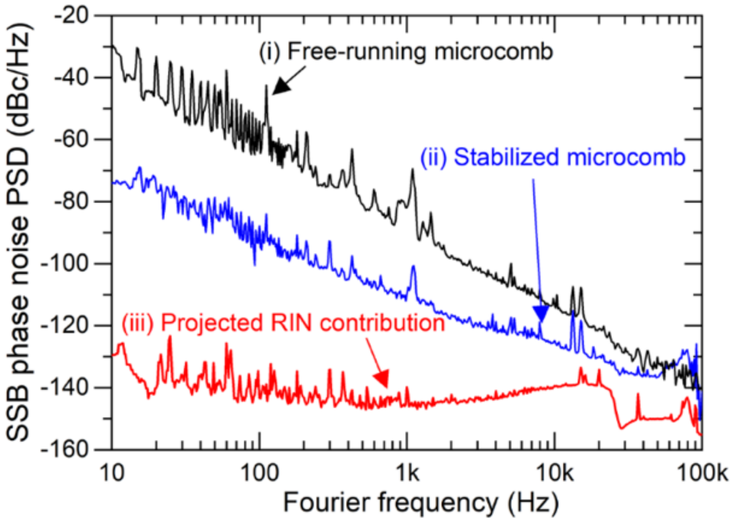

Supplementary Figure 9 | Impact of RIN-converted phase noise. (i) Free-running microcomb. (ii) Stabilized microcomb. (iii) Projected RIN-converted phase noise with APC coefficient of 8 rad/% in photodetection.

Thermal noise of optical fibre in repetition-rate stabilization. For shorter than 0.1 s time scale, the stabilized performance is limited by the white frequency noise of the interference signal, which is higher than the thermomechanical and thermoconductive noise. Note that the projected thermal noise (curve (iii) in Fig. 3**a** of the main manuscript) includes the thermomechanical length fluctuation[12] and thermoconductive length fluctuation[13]. We compute the thermal noise-induced phase error in the 1-km-long (which is 2 km for the round-trip) fibre interferometer ($S_{\phi_{thermal}}(f)$) using refs. 12 and 13. This phase error is converted to the laser frequency noise as $S_{\nu}(f) = \left(\frac{1}{\tau}\right)^2 S_{\phi_{thermal}}(f)$, where τ is the timing delay induced by the interferometer. In this experiment, the common-mode f_{ceo} noise is rejected so that the laser frequency noise ($S_{\nu}(f)$) corresponds to 2.5 THz carrier (i.e., $(m\text{-}n)f_{rep}$). Finally, the frequency noise of 2.5 THz is converted to the phase noise of 22 GHz.

Relatively large thermo-optic coefficient of fibre (~10^{-6} K^{-1}) can cause timing drift, which is also observed in the Allan deviation (curve (iii) of Fig. 3**c**) after ~0.4 s averaging time. To

minimize the impact of acoustic noise and thermal drift coupled to the fibre delay-line, we packaged the fibre-stabilizer in an air-tight box to isolate it from the laboratory environment (see photo in Fig. 1**a**). Although we did not use a heavy piece of metal or special material with large thermal constant to keep the stabilization system compact, we found that this air-tight box works fairly well. Also note that we performed the measurement of noise and stability after waiting for several hours from turning on the system, so that the entire electronics, fibre and micro-comb systems reach a fairly thermal-equilibrium state.

Supplementary Note 4: Timing jitter and phase noise measurement methods

Optical-domain timing jitter characterization of the free-running comb. For timing jitter characterization of free-running combs, a 60-m-long PZT stretcher is used for the time delay (τ~300 ns) in a Mach-Zehnder interferometer (Type 1 in Supplementary Fig. 10). The interferometer output is split into ν_m and ν_n by the WDM. The carrier of the photodetected signals is f_{AO} due to the frequency shift by the $AOFS_2$ in the delay arm. The phase noise of each photodetected signal at f_{AO} contains the frequency noise of each comb mode weighted by the time delay (τ), in the form of $\delta[\tau\times(mf_{rep}+f_{ceo}+f_{AO})]$ and $\delta[\tau\times(nf_{rep}+f_{ceo}+f_{AO})]$. Each photodetected signal is filtered by an RF bandpass filter centred at f_{AO}, amplified, and then down-converted to the baseband by a frequency mixer. The resulting low-pass filtered mixer output contains the repetition frequency noise in the form of $\delta[\tau\times(m\text{-}n)f_{rep}]$, where $(m\text{-}n)f_{rep}$ corresponds to the frequency difference between the two comb modes (~2.5 THz). The delay-locked loop (DLL) is formed by applying $\delta[\tau\times(m\text{-}n)f_{rep}]$ signal to the PZT stretcher in the Mach-Zehnder interferometer to avoid drift during the phase noise measurement. In this experiment, the locking bandwidth is ~300 Hz. The repetition-rate frequency noise above the locking bandwidth is measured by signal analysers and converted to the repetition-rate phase noise. The repetition-rate phase noise of the free-running microcomb is measured by this method

(curve (i) in Fig. 2**c**).

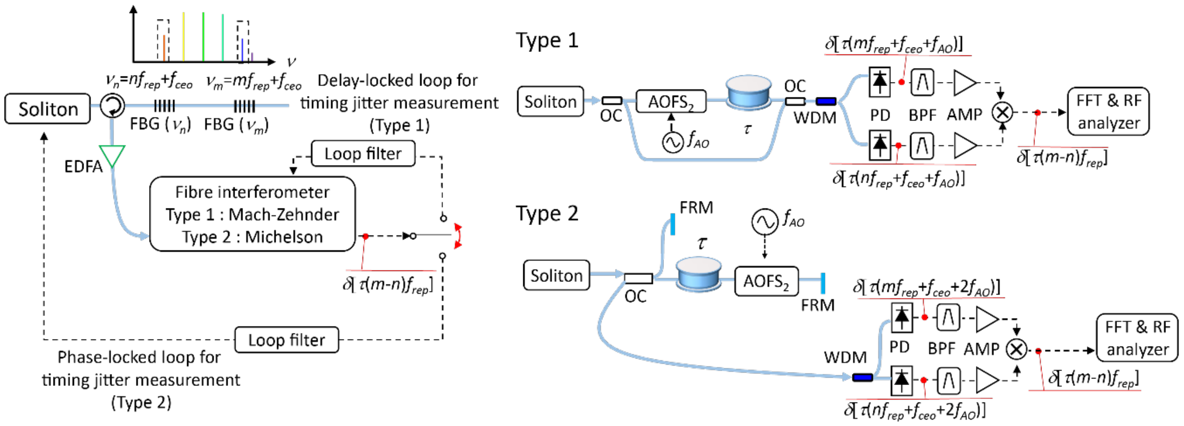

Supplementary Figure 10 | Repetition-rate phase noise (timing jitter) measurement and stabilization using a fibre delayline-based method. Experimental setup based on a Mach-Zehnder interferometer (Type 1) and a Michelson interferometer (Type 2). FBG, fibre-Bragg grating; f_{rep}, repetition rate; f_{ceo}, carrier-envelope offset frequency; m and n, mode numbers for comb modes; EDFA, Er-doped fibre amplifier; AOFS$_2$, acousto-optic frequency shifter; f_{AO}, driving frequency for the AOFS; τ, time delay induced by a PZT stretcher (Type 1) or a fibre link (Type 2); FRM, Faraday rotating mirror; OC, optical coupler; WDM, wavelength-division multiplexing coupler; PD, photodetector; BPF, RF bandpass filter; AMP, RF amplifier.

Note that the functions of Mach-Zehnder and Michelson interferometers are exactly same and is interchangeable. In this work, the reason why we used the Mach-Zehnder interferometer for free-running jitter measurement was to obtain twice more measurement bandwidth than Michelson interferometer when using the same fibre delay-line spool. In this case, although the measurement sensitivity becomes twice worse, it did not limit the measurement result of free-running combs, so we could measure the jitter over broader offset frequency range.

Optical-domain timing jitter characterization of the stabilized microcomb. The repetition-rate phase noise (timing jitter) of the fibre-stabilized microcomb is measured by an independent

fibre-Michelson interferometer in the optical domain and is compared with the microwave phase noise measurement result. The independent interferometer (out-of-loop interferometer in Supplementary Fig. 11**a**) has an identical configuration to the interferometer for the stabilization (in-loop interferometer in Supplementary Fig. 11**a**). Since the phase noise measurement method used in Supplementary Fig. 10 (which uses a 60-m-long fibre link) has a limited detection sensitivity to measure the phase noise of the 1 km-long-fibre-stabilized microcomb, an identical length of fibre link (1 km) is used for characterizing the fibre-stabilized comb. As expected, the repetition-rate phase noise measured in the optical domain (curve (i) in Supplementary Fig. 11**b**) shows a good agreement with the microwave phase noise result (curve (ii) in Supplementary Fig. 11**b**).

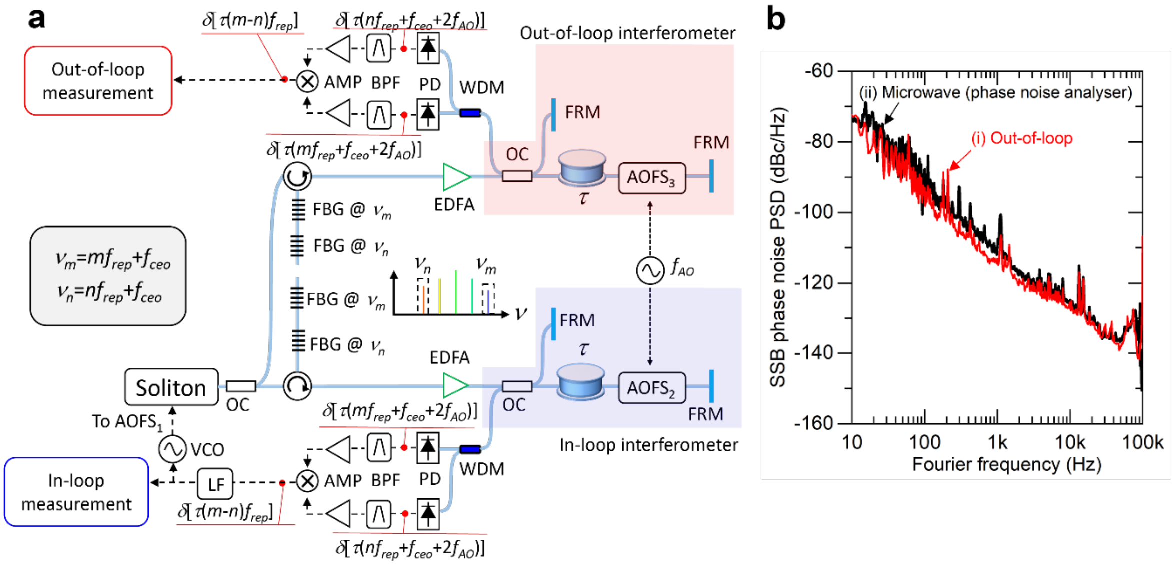

Supplementary Figure 11 | Experimental setup and results of optical-domain phase-noise characterization of the fibre-stabilized microcomb. a, Experimental setup. An in-loop interferometer is used to stabilize the repetition rate phase noise, and an out-of-loop interferometer is used to measure the phase noise of the fibre-stabilized microcomb. LF, loop filter; **b,** Single-sideband (SSB) phase noise power spectral densities (PSDs) of the fibre-stabilized microcomb scaled to the 22-GHz carrier. (i) Measured phase noise by an out-of-loop interferometer. (ii) Measured microwave phase noise by the phase noise analyser (same as curve (ii) in Fig. 3**a**). A 1-km-long fibre link is used for both cases.

Frequency measurement of the microwave beat note. Two fibre-stabilized silica microcombs generate a microwave beat note to measure the frequency fluctuation with high sensitivity. Microwaves from each microcomb (22.059 GHz and 22.083 GHz) are extracted by two MUTC-photodiodes (FP1015a from Freedom Photonics) with -8 V bias voltage. The photocurrent and the RF power extracted from each MUTC-PD are ~7 mA and -2 dBm, respectively. Each microwave signal is filtered by a bandpass filter centred at 22 GHz and amplified by an RF amplifier up to +10 dBm. The frequency of the down-converted microwave beat note obtained at the frequency mixer output is 23.3 MHz. The frequency fluctuation is measured by a commercial high-sensitivity digital cross-correlator[14] (5125A, Microsemi) referenced by a 10-MHz ultralow phase noise oven-controlled crystal oscillator.

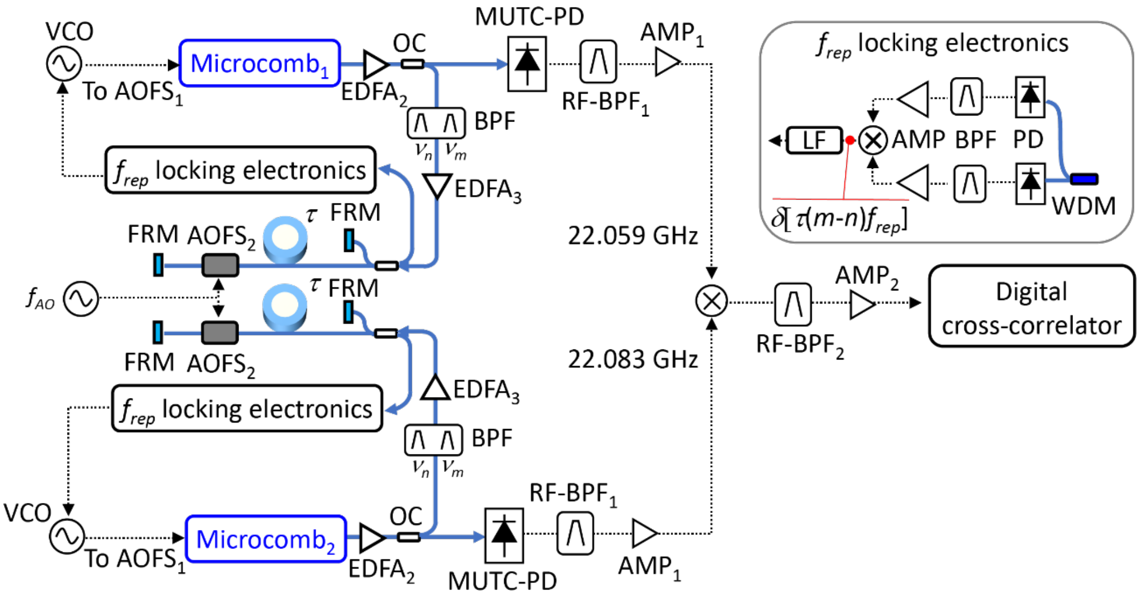

Supplementary Figure 12 | Measurement schematic of the frequency fluctuation. Frequency fluctuation of the down-converted microwave beat note is measured. MUTC-PD, modified uni-travelling carrier photodiode; $RF\text{-}BPF_1$, RF bandpass filter centred at 22 GHz; AMP_1, RF amplifier operating at 22 GHz; $RF\text{-}BPF_2$, RF bandpass filter for the microwave beat note; AMP_2, RF amplifier operating at 23.3 MHz.

Supplementary References

1. Karpov, M. *et al.* Raman self-frequency shift of dissipative Kerr solitons in an optical microresonator. *Phys. Rev. Lett.* **116,** 103902 (2016).
2. Akhmediev, N. & Karlsson, M. Cherenkov radiation emitted by solitons in optical fibers. *Phys. Rev. A* **51,** 2602-2607 (1995).
3. Yi, X. *et al.* Single-mode dispersive waves and soliton microcomb dynamics. *Nat. Commun.* **8,** 14869 (2017).
4. Herr, T. *et al.* Temporal solitons in optical microresonators. *Nat. Photonics* **8,** 145–152 (2014).
5. Kéfélian, F., Jiang, H., Lemonde, P. & Santarelli, G. Ultralow-frequency-noise stabilization of a laser by locking to an optical fiber-delay line. *Opt. Lett.* **34,** 914-916 (2009).
6. Matsko, A. B. & Maleki, L. On timing jitter of mode locked Kerr frequency combs. *Opt. Express* **21,** 28862–28876 (2013).
7. Bao, C. *et al.* Quantum diffusion of microcavity solitons. *Nat. Phys.* **17,** 462-466 (2021).
8. Huang, G. *et al.* Thermorefractive noise in silicon-nitride microresonators. *Phys. Rev. A* **99,** 061801 (2019).
9. Yang, Q. F. *et al.* Dispersive-wave induced noise limits in miniature soliton microwave sources. *Nat. Commun.* **12,** 1442 (2021).
10. Cahill, J. P., Okusaga, O., Zhou, W., Menyuk, C. R. & Carter, G. M. Superlinear growth of Rayleigh scattering-induced intensity noise in single-mode fibers. *Opt. Express* **23,** 6400–6407 (2015).
11. Taylor, J. *et al.* Characterization of power-to-phase conversion in high-speed p-i-n photodiodes, *IEEE Photon. J.* **3,** 140-151 (2011).
12. Duan, L. Z. Intrinsic thermal noise of optical fibres due to mechanical dissipation.

Electron. Lett. **46,** 1515–1516 (2010).

13. Duan, L. General treatment of the thermal noises in optical fibers. *Phys. Rev. A* **86,** 023817 (2012).
14. Nelson, C. W. & Howe, D. A. A sub-sampling digital PM/AM noise measurement system. In *Proceedings of the IEEE Frequency Control Symposium*. 1-4 (IEEE, 2012).
